# *catena*-Poly[[[diaqua­bis(8-hydroxy­quinoline *N*-oxide-κ*O* ^1^)cobalt(II)]-μ-2,5-dimethyl­pyrazine 1,4-dioxide-κ^2^ *O* ^1^:*O* ^4^] bis­(perchlorate)]

**DOI:** 10.1107/S1600536810008895

**Published:** 2010-03-13

**Authors:** Li Qin Gu, Jin Min Li

**Affiliations:** a School of Chemistry and Chemical Engineering, Shanxi Datong University, Datong 037009, People’s Republic of China

## Abstract

In the title complex, {[Co(C_6_H_8_N_2_O_2_)(C_9_H_7_NO_2_)(H_2_O)_2_](ClO_4_)_2_}_*n*_, the Co^II^ ion lies on an inversion centre and is coordinated in a slightly distorted octa­hedral environment. The 2,5-dimethyl­pyrazine 1,4-dioxide ligand, which also lies on an inversion center, acts as a bridging ligand, linking symmetry-related Co^II^ ions [Co⋯Co = 8.669 (3) Å] and forming one-dimensional chains along the *b* axis. In the crystal structure, these chains are linked by inter­molecular aqua–perchlorate O—H⋯O hydrogen bonds, forming two-dimensional layers which are in turn connected into a three-dimensional network *via* π–π stacking inter­actions between quinoline rings, with a centroid–centroid distance of 3.580 (3) Å. An intermolecular O—H⋯Cl inter­action is also present.

## Related literature

For the isostructural Mn(II) complex, see:, see: Shi *et al.* (2009 ▶).
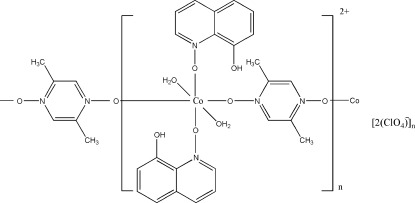


## Experimental

### 

#### Crystal data

[Co(C_6_H_8_N_2_O_2_)(C_9_H_7_NO_2_)(H_2_O)_2_](ClO_4_)_2_
*M* *_r_* = 756.32
Triclinic, 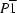

*a* = 8.530 (3) Å
*b* = 8.669 (3) Å
*c* = 11.182 (3) Å
α = 84.319 (4)°
β = 86.077 (4)°
γ = 63.475 (4)°
*V* = 735.9 (4) Å^3^
*Z* = 1
Mo *K*α radiation
μ = 0.85 mm^−1^
*T* = 298 K
0.44 × 0.37 × 0.17 mm

#### Data collection

Bruker SMART APEX CCD diffractometer
Absorption correction: multi-scan (*SADABS*; Sheldrick, 1996 ▶) *T*_min_ = 0.706, *T*_max_ = 0.869
3772 measured reflections
2617 independent reflections
2267 reflections with *I* > 2σ(*I*)
*R*_int_ = 0.022

#### Refinement

*R*[*F* ^2^ > 2σ(*F* ^2^)] = 0.063
*wR*(*F* ^2^) = 0.184
*S* = 1.06
2617 reflections
215 parameters
H-atom parameters constrained
Δρ_max_ = 0.89 e Å^−3^
Δρ_min_ = −0.60 e Å^−3^

### 

Data collection: *SMART* (Bruker, 1997 ▶); cell refinement: *SAINT* (Bruker, 1997 ▶); data reduction: *SAINT*; program(s) used to solve structure: *SHELXTL* (Sheldrick, 2008 ▶); program(s) used to refine structure: *SHELXTL*; molecular graphics: *SHELXTL*; software used to prepare material for publication: *SHELXTL*.

## Supplementary Material

Crystal structure: contains datablocks I, global. DOI: 10.1107/S1600536810008895/lh5007sup1.cif

Structure factors: contains datablocks I. DOI: 10.1107/S1600536810008895/lh5007Isup2.hkl

Additional supplementary materials:  crystallographic information; 3D view; checkCIF report

## Figures and Tables

**Table 1:** Hydrogen-bond geometry (Å, °)

*D*—H⋯*A*	*D*—H	H⋯*A*	*D*⋯*A*	*D*—H⋯*A*
O3—H9⋯O7^i^	0.84	2.59	3.324 (15)	146
O3—H9⋯O5^i^	0.84	2.42	3.219 (16)	159
O3—H8⋯O5^ii^	0.84	2.63	3.234 (13)	130
O3—H8⋯O6^ii^	0.84	2.14	2.970 (7)	166
O3—H8⋯Cl1^ii^	0.84	2.95	3.739 (3)	155

Symmetry codes: (i) 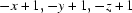; (ii) 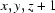.

**Fig. 1.**
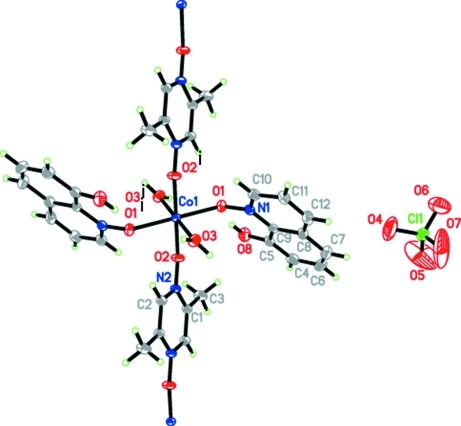
View of coordination environment in (I), showing the the atom numbering scheme with displacement ellipsoids drawn at the 30% probability level; Symmetry code: (i) -x+2, -y, -z+2.

**Fig. 2.**
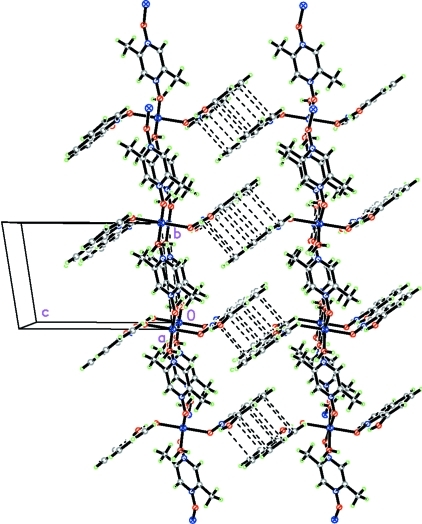
Part of the crystal structure showing π–π stacking interaction (dashed lines) between adjacent two-dimensional layers.

## supplementary crystallographic information

### Comment

The derivatives of pyrazine 1,4-dioxide and 8-hydroxyquinoline N-oxide play vital roles in a modern coordination chemistry and the isostructural Mn(II) complex of the title compound has already been published (Shi *et al.*, 2009). Our interest in designing new complexes led to us obtaining the title complex, (I), and we report the crystal structure herein.

The coordination geometry of Co^II^ ion in (I) is shown in Fig. 1. The Co^II^ ion is located on inversion centre and assumes a slightly distorted octahedral CoO_6_ coordination geometry. The centrosymmetric pyrazine 1,4-dioxide ligand functions as bridging connecting two symmetry related Co^II^ ions with a separation of 8.669 (3) Å, forming one-dimensional chains along the b axis. In the crystal structure, these chains are linked by intermolecular O-H_aqua_···O_perchlorate_ hydrogen bonds to form two-dimensional layers. In addition, there are π–π stacking interactions (Fig. 2) between neighbouring layers involving quilinoline rings with relevant distances being Cg1···Cg2^i^ = 3.580 (3) Å and Cg1···Cg2^i^_perp_ = 3.410 Å and α = 3.19° [symmetry code (i) 2-*x*, -*y*, 1-*z*; Cg1 and Cg2 are the centroids of C8—C12/N1 ring and C4—C9 ring, respectively; Cg1···Cg2^i^_perp_ is the perpendicular distance from ring Cg1 to ring Cg2^i^; α is the dihedral angle between ring plane Cg1 and ring plane Cg2^i^].

### Experimental

10 ml ethanol solution of 8-hydroxyquinoline N-oxide (0.2015 g, 1.25 mmol) was added into 10 ml of aqueous solution containing hydrated cobalt perchlorate (0.2291 g, 0.626 mmol) and 2,5-dimethylpyrazine 1,4-dioxide (0.0895 g, 0.639 mmol). The resulting solution was stirred for a few minutes. The red single crystals were obtained from the filtrate by slow evaporation for about three weeks.

### Refinement

H atoms from hydroxy and water molecule were found in difference Fourier maps and fixed with O—H = 0.84 Å; other H atoms were placed in calculated positions with C—H = 0.93-0.97 Å. All H atoms were refined as riding, with U_iso_(H) = 1.5_eq_(O) for hydroxy group and water molecule, and U_iso_(H) = 1.5 U_eq_(C) for methyl group and U_iso_(H) = 1.2 U_eq_(C) for other groups.

### Crystal data

**Table:**

[Co(C_6_H_8_N_2_O_2_)(C_9_H_7_NO_2_)(H_2_O)_2_](ClO_4_)_2_	*Z* = 1
*M**_r_* = 756.32	*F*(000) = 387
Triclinic, *P*1	*D*_x_ = 1.707 Mg m^−^^3^
Hall symbol: -P 1	Mo *K*α radiation, λ = 0.71073 Å
*a* = 8.530 (3) Å	Cell parameters from 1924 reflections
*b* = 8.669 (3) Å	θ = 2.6–27.6°
*c* = 11.182 (3) Å	µ = 0.85 mm^−^^1^
α = 84.319 (4)°	*T* = 298 K
β = 86.077 (4)°	Block, red
γ = 63.475 (4)°	0.44 × 0.37 × 0.17 mm
*V* = 735.9 (4) Å^3^	

### Data collection

**Table:**

Bruker SMART APEX CCD diffractometer	2617 independent reflections
Radiation source: fine-focus sealed tube	2267 reflections with *I* > 2σ(*I*)
graphite	*R*_int_ = 0.022
φ and ω scans	θ_max_ = 25.3°, θ_min_ = 1.8°
Absorption correction: multi-scan (*SADABS*; Sheldrick, 1996)	*h* = −10→10
*T*_min_ = 0.706, *T*_max_ = 0.869	*k* = −9→10
3772 measured reflections	*l* = −13→9

### Refinement

**Table:**

Refinement on *F*^2^	Primary atom site location: structure-invariant direct methods
Least-squares matrix: full	Secondary atom site location: difference Fourier map
*R*[*F*^2^ > 2σ(*F*^2^)] = 0.063	Hydrogen site location: inferred from neighbouring sites
*wR*(*F*^2^) = 0.184	H-atom parameters constrained
*S* = 1.06	*w* = 1/[σ^2^(*F*_o_^2^) + (0.1181*P*)^2^ + 0.9504*P*] where *P* = (*F*_o_^2^ + 2*F*_c_^2^)/3
2617 reflections	(Δ/σ)_max_ = 0.001
215 parameters	Δρ_max_ = 0.89 e Å^−^^3^
0 restraints	Δρ_min_ = −0.60 e Å^−^^3^

### Special details

**Table:**

Geometry. All esds (except the esd in the dihedral angle between two l.s. planes) are estimated using the full covariance matrix. The cell esds are taken into account individually in the estimation of esds in distances, angles and torsion angles; correlations between esds in cell parameters are only used when they are defined by crystal symmetry. An approximate (isotropic) treatment of cell esds is used for estimating esds involving l.s. planes.
Refinement. Refinement of *F*^2^ against ALL reflections. The weighted *R*-factor wR and goodness of fit *S* are based on *F*^2^, conventional *R*-factors *R* are based on *F*, with *F* set to zero for negative *F*^2^. The threshold expression of *F*^2^ > σ(*F*^2^) is used only for calculating *R*-factors(gt) etc. and is not relevant to the choice of reflections for refinement. *R*-factors based on *F*^2^ are statistically about twice as large as those based on *F*, and *R*- factors based on ALL data will be even larger.

### Fractional atomic coordinates and isotropic or equivalent isotropic displacement parameters (Å^2^)

**Table:**

	*x*	*y*	*z*	*U*_iso_*/*U*_eq_	
C1	1.1372 (5)	0.4195 (5)	0.9207 (3)	0.0297 (8)	
C2	0.9253 (6)	0.4091 (5)	1.0618 (3)	0.0315 (9)	
H2	0.8746	0.3450	1.1054	0.038*	
C3	1.2850 (6)	0.3280 (6)	0.8383 (4)	0.0444 (11)	
H3A	1.3785	0.2370	0.8829	0.067*	
H3B	1.3256	0.4077	0.7992	0.067*	
H3C	1.2475	0.2797	0.7790	0.067*	
C4	1.0908 (7)	0.2478 (7)	0.5122 (4)	0.0484 (12)	
H4	1.1911	0.2641	0.4964	0.058*	
C5	1.0843 (6)	0.1442 (6)	0.6116 (4)	0.0346 (9)	
C6	0.9521 (8)	0.3287 (7)	0.4349 (4)	0.0539 (13)	
H6	0.9609	0.3976	0.3681	0.065*	
C7	0.8036 (8)	0.3083 (7)	0.4557 (4)	0.0542 (13)	
H7	0.7117	0.3632	0.4029	0.065*	
C8	0.7873 (6)	0.2052 (6)	0.5563 (4)	0.0385 (10)	
C9	0.9281 (6)	0.1257 (5)	0.6372 (3)	0.0311 (9)	
C10	0.7684 (6)	−0.0009 (6)	0.7554 (4)	0.0372 (10)	
H10	0.7620	−0.0699	0.8229	0.045*	
C11	0.6310 (6)	0.0691 (6)	0.6755 (4)	0.0402 (10)	
H11	0.5354	0.0443	0.6879	0.048*	
C12	0.6390 (6)	0.1744 (7)	0.5792 (4)	0.0450 (11)	
H12	0.5451	0.2268	0.5278	0.054*	
Cl1	0.38256 (16)	0.38330 (16)	0.21191 (10)	0.0453 (4)	
Co1	1.0000	0.0000	1.0000	0.0255 (3)	
N1	0.9070 (4)	0.0284 (4)	0.7373 (3)	0.0294 (7)	
N2	1.0584 (4)	0.3301 (4)	0.9833 (3)	0.0272 (7)	
O1	1.0368 (4)	−0.0431 (4)	0.8181 (2)	0.0336 (7)	
O2	1.1177 (4)	0.1644 (4)	0.9666 (3)	0.0362 (7)	
O3	0.7397 (4)	0.1908 (4)	0.9808 (3)	0.0399 (7)	
H8	0.6631	0.2000	1.0353	0.060*	
H9	0.7058	0.2760	0.9292	0.060*	
O4	0.4024 (10)	0.3329 (9)	0.3327 (5)	0.124 (2)	
O5	0.4894 (19)	0.4538 (19)	0.1748 (10)	0.250 (7)	
O6	0.4261 (10)	0.2460 (8)	0.1370 (6)	0.116 (2)	
O7	0.2142 (12)	0.5000 (16)	0.1965 (7)	0.235 (7)	
O8	1.2287 (4)	0.0584 (5)	0.6787 (3)	0.0483 (8)	
H1	1.1957	0.0109	0.7386	0.072*	

### Atomic displacement parameters (Å^2^)

**Table:**

	*U*^11^	*U*^22^	*U*^33^	*U*^12^	*U*^13^	*U*^23^
C1	0.035 (2)	0.029 (2)	0.0296 (19)	−0.0179 (18)	0.0033 (16)	−0.0039 (15)
C2	0.040 (2)	0.033 (2)	0.029 (2)	−0.0230 (19)	0.0056 (16)	−0.0033 (16)
C3	0.050 (3)	0.040 (3)	0.050 (3)	−0.026 (2)	0.019 (2)	−0.015 (2)
C4	0.063 (3)	0.050 (3)	0.043 (3)	−0.036 (3)	0.011 (2)	−0.010 (2)
C5	0.040 (2)	0.037 (2)	0.031 (2)	−0.0203 (19)	0.0026 (17)	−0.0093 (17)
C6	0.081 (4)	0.045 (3)	0.038 (3)	−0.032 (3)	0.004 (2)	0.005 (2)
C7	0.067 (4)	0.054 (3)	0.037 (3)	−0.024 (3)	−0.008 (2)	0.006 (2)
C8	0.042 (2)	0.038 (2)	0.033 (2)	−0.014 (2)	−0.0034 (18)	−0.0055 (18)
C9	0.042 (2)	0.027 (2)	0.0264 (19)	−0.0159 (18)	0.0032 (16)	−0.0081 (15)
C10	0.041 (2)	0.040 (2)	0.035 (2)	−0.022 (2)	0.0060 (18)	−0.0096 (18)
C11	0.035 (2)	0.050 (3)	0.039 (2)	−0.021 (2)	0.0050 (18)	−0.014 (2)
C12	0.039 (3)	0.051 (3)	0.038 (2)	−0.012 (2)	−0.0062 (19)	−0.008 (2)
Cl1	0.0507 (7)	0.0481 (7)	0.0414 (6)	−0.0273 (6)	−0.0088 (5)	0.0098 (5)
Co1	0.0320 (4)	0.0199 (4)	0.0251 (4)	−0.0122 (3)	−0.0005 (3)	−0.0010 (3)
N1	0.0329 (18)	0.0289 (17)	0.0272 (16)	−0.0136 (15)	0.0010 (13)	−0.0068 (13)
N2	0.0329 (18)	0.0207 (16)	0.0309 (17)	−0.0143 (14)	0.0018 (13)	−0.0045 (13)
O1	0.0326 (15)	0.0389 (16)	0.0279 (14)	−0.0145 (13)	−0.0049 (11)	0.0006 (12)
O2	0.0453 (17)	0.0230 (14)	0.0449 (17)	−0.0197 (13)	0.0127 (13)	−0.0097 (12)
O3	0.0368 (17)	0.0344 (16)	0.0402 (17)	−0.0095 (14)	−0.0003 (13)	0.0018 (13)
O4	0.156 (6)	0.124 (5)	0.059 (3)	−0.036 (4)	−0.032 (3)	0.028 (3)
O5	0.369 (17)	0.364 (17)	0.208 (11)	−0.334 (16)	0.114 (10)	−0.095 (11)
O6	0.180 (7)	0.107 (4)	0.100 (4)	−0.098 (5)	0.025 (4)	−0.029 (3)
O7	0.135 (7)	0.290 (12)	0.109 (5)	0.074 (7)	−0.065 (5)	−0.057 (7)
O8	0.0461 (19)	0.068 (2)	0.0434 (18)	−0.0375 (18)	−0.0012 (14)	0.0017 (16)

### Geometric parameters (Å, °)

**Table:**

C1—N2	1.355 (5)	C10—N1	1.315 (6)
C1—C2^i^	1.367 (6)	C10—C11	1.392 (6)
C1—C3	1.470 (6)	C10—H10	0.9300
C2—N2	1.348 (5)	C11—C12	1.360 (7)
C2—C1^i^	1.367 (6)	C11—H11	0.9300
C2—H2	0.9300	C12—H12	0.9300
C3—H3A	0.9600	Cl1—O5	1.330 (7)
C3—H3B	0.9600	Cl1—O7	1.348 (7)
C3—H3C	0.9600	Cl1—O4	1.376 (5)
C4—C5	1.372 (6)	Cl1—O6	1.419 (6)
C4—C6	1.385 (8)	Co1—O2	2.074 (3)
C4—H4	0.9300	Co1—O2^ii^	2.074 (3)
C5—O8	1.353 (5)	Co1—O1^ii^	2.082 (3)
C5—C9	1.418 (6)	Co1—O1	2.082 (3)
C6—C7	1.357 (8)	Co1—O3^ii^	2.104 (3)
C6—H6	0.9300	Co1—O3	2.104 (3)
C7—C8	1.405 (7)	N1—O1	1.357 (4)
C7—H7	0.9300	N2—O2	1.321 (4)
C8—C12	1.407 (7)	O3—H8	0.8448
C8—C9	1.422 (6)	O3—H9	0.8414
C9—N1	1.385 (5)	O8—H1	0.8483
			
N2—C1—C2^i^	117.9 (4)	C11—C12—C8	120.9 (4)
N2—C1—C3	118.7 (4)	C11—C12—H12	119.5
C2^i^—C1—C3	123.4 (4)	C8—C12—H12	119.5
N2—C2—C1^i^	121.9 (4)	O5—Cl1—O7	110.3 (9)
N2—C2—H2	119.1	O5—Cl1—O4	110.0 (7)
C1^i^—C2—H2	119.1	O7—Cl1—O4	107.4 (5)
C1—C3—H3A	109.5	O5—Cl1—O6	105.0 (6)
C1—C3—H3B	109.5	O7—Cl1—O6	109.8 (6)
H3A—C3—H3B	109.5	O4—Cl1—O6	114.4 (4)
C1—C3—H3C	109.5	O2—Co1—O2^ii^	180.000 (1)
H3A—C3—H3C	109.5	O2—Co1—O1^ii^	92.05 (12)
H3B—C3—H3C	109.5	O2^ii^—Co1—O1^ii^	87.95 (12)
C5—C4—C6	121.8 (5)	O2—Co1—O1	87.95 (12)
C5—C4—H4	119.1	O2^ii^—Co1—O1	92.05 (12)
C6—C4—H4	119.1	O1^ii^—Co1—O1	180.000 (1)
O8—C5—C4	119.0 (4)	O2—Co1—O3^ii^	83.25 (12)
O8—C5—C9	122.4 (4)	O2^ii^—Co1—O3^ii^	96.75 (12)
C4—C5—C9	118.5 (4)	O1^ii^—Co1—O3^ii^	93.87 (12)
C7—C6—C4	120.6 (5)	O1—Co1—O3^ii^	86.13 (12)
C7—C6—H6	119.7	O2—Co1—O3	96.75 (12)
C4—C6—H6	119.7	O2^ii^—Co1—O3	83.25 (12)
C6—C7—C8	120.7 (5)	O1^ii^—Co1—O3	86.13 (12)
C6—C7—H7	119.7	O1—Co1—O3	93.87 (12)
C8—C7—H7	119.7	O3^ii^—Co1—O3	180.000 (1)
C7—C8—C12	122.8 (4)	C10—N1—O1	118.8 (3)
C7—C8—C9	118.6 (4)	C10—N1—C9	122.6 (4)
C12—C8—C9	118.6 (4)	O1—N1—C9	118.5 (3)
N1—C9—C5	122.8 (4)	O2—N2—C2	121.6 (3)
N1—C9—C8	117.5 (4)	O2—N2—C1	118.2 (3)
C5—C9—C8	119.7 (4)	C2—N2—C1	120.2 (3)
N1—C10—C11	121.4 (4)	N1—O1—Co1	123.9 (2)
N1—C10—H10	119.3	N2—O2—Co1	130.8 (2)
C11—C10—H10	119.3	Co1—O3—H8	120.6
C12—C11—C10	118.8 (4)	Co1—O3—H9	125.3
C12—C11—H11	120.6	H8—O3—H9	112.8
C10—C11—H11	120.6	C5—O8—H1	104.7
			
C6—C4—C5—O8	175.1 (4)	C5—C9—N1—O1	4.2 (5)
C6—C4—C5—C9	−2.3 (7)	C8—C9—N1—O1	−177.5 (3)
C5—C4—C6—C7	0.3 (8)	C1^i^—C2—N2—O2	−179.8 (4)
C4—C6—C7—C8	0.2 (8)	C1^i^—C2—N2—C1	1.5 (7)
C6—C7—C8—C12	−177.4 (5)	C2^i^—C1—N2—O2	179.8 (3)
C6—C7—C8—C9	1.3 (7)	C3—C1—N2—O2	−0.4 (6)
O8—C5—C9—N1	4.7 (6)	C2^i^—C1—N2—C2	−1.5 (6)
C4—C5—C9—N1	−178.0 (4)	C3—C1—N2—C2	178.3 (4)
O8—C5—C9—C8	−173.5 (4)	C10—N1—O1—Co1	−58.2 (4)
C4—C5—C9—C8	3.8 (6)	C9—N1—O1—Co1	123.5 (3)
C7—C8—C9—N1	178.3 (4)	O2—Co1—O1—N1	−103.8 (3)
C12—C8—C9—N1	−2.9 (6)	O2^ii^—Co1—O1—N1	76.2 (3)
C7—C8—C9—C5	−3.3 (6)	O1^ii^—Co1—O1—N1	−52 (100)
C12—C8—C9—C5	175.4 (4)	O3^ii^—Co1—O1—N1	172.8 (3)
N1—C10—C11—C12	−2.2 (7)	O3—Co1—O1—N1	−7.2 (3)
C10—C11—C12—C8	3.4 (7)	C2—N2—O2—Co1	21.7 (5)
C7—C8—C12—C11	177.8 (5)	C1—N2—O2—Co1	−159.6 (3)
C9—C8—C12—C11	−0.9 (7)	O2^ii^—Co1—O2—N2	22 (100)
C11—C10—N1—O1	180.0 (4)	O1^ii^—Co1—O2—N2	−58.9 (3)
C11—C10—N1—C9	−1.9 (6)	O1—Co1—O2—N2	121.1 (3)
C5—C9—N1—C10	−173.9 (4)	O3^ii^—Co1—O2—N2	−152.5 (3)
C8—C9—N1—C10	4.4 (6)	O3—Co1—O2—N2	27.5 (3)

Symmetry codes: (i) −*x*+2, −*y*+1, −*z*+2; (ii) −*x*+2, −*y*, −*z*+2.

### Hydrogen-bond geometry (Å, °)

**Table:**

*D*—H···*A*	*D*—H	H···*A*	*D*···*A*	*D*—H···*A*
O3—H9···O7^iii^	0.84	2.59	3.324 (15)	146
O3—H9···O5^iii^	0.84	2.42	3.219 (16)	159
O3—H8···O5^iv^	0.84	2.63	3.234 (13)	130
O3—H8···O6^iv^	0.84	2.14	2.970 (7)	166
O3—H8···Cl1^iv^	0.84	2.95	3.739 (3)	155

Symmetry codes: (iii) −*x*+1, −*y*+1, −*z*+1; (iv) *x*, *y*, *z*+1.
